# Combination therapy of tyrosine kinase inhibitor sorafenib with the HSP90 inhibitor onalespib as a novel treatment regimen for thyroid cancer

**DOI:** 10.1038/s41598-023-43486-z

**Published:** 2023-10-06

**Authors:** Anja Charlotte Lundgren Mortensen, Hanna Berglund, Mehran Hariri, Eleftherios Papalanis, Christer Malmberg, Diana Spiegelberg

**Affiliations:** 1 Department of Immunology, Genetics and Pathology, Uppsala University, Uppsala, Sweden; 2 Department of Molecular Medicine and Surgery, Karolinska Institutet, Stockholm, Sweden; 3 Department of Medical Sciences, Uppsala University, Uppsala, Sweden; 4 Department of Surgical Sciences, Uppsala University, Uppsala, Sweden

**Keywords:** Endocrine cancer, Targeted therapies

## Abstract

Thyroid cancer is the most common endocrine malignancy, affecting nearly 600,000 new patients worldwide. Treatment with the BRAF inhibitor sorafenib partially prolongs progression-free survival in thyroid cancer patients, but fails to improve overall survival. This study examines enhancing sorafenib efficacy by combination therapy with the novel HSP90 inhibitor onalespib. In vitro efficacy of sorafenib and onalespib monotherapy as well as in combination was assessed in papillary (PTC) and anaplastic (ATC) thyroid cancer cells using cell viability and colony formation assays. Migration potential was studied in wound healing assays. The in vivo efficacy of sorafenib and onalespib therapy was evaluated in mice bearing BHT-101 xenografts. Sorafenib in combination with onalespib significantly inhibited PTC and ATC cell proliferation, decreased metabolic activity and cancer cell migration. In addition, the drug combination approach significantly inhibited tumor growth in the xenograft model and prolonged the median survival. Our results suggest that combination therapy with sorafenib and onalespib could be used as a new therapeutic approach in the treatment of thyroid cancer, significantly improving the results obtained with sorafenib as monotherapy. This approach has the potential to reduce treatment adaptation while at the same time providing therapeutic anti-cancer benefits such as reducing tumor growth and metastatic potential.

## Introduction

Thyroid cancer is the most common endocrine malignancy, affecting nearly 600,000 new patients worldwide, resulting in more than 43,000 deaths each year^1^. Women are four times more likely to be diagnosed with thyroid cancer than men and it is the seventh most common cancer among women^2^.

Thyroid cancer is comprised of several cancers, which arise from either the follicular cells (follicular (FTC), papillary (PTC) and anaplastic (ATC) thyroid carcinoma) or the parafollicular cells (medullary thyroid carcinoma, MTC)^3^. The vast majority of PTC and FTC cases are well-differentiated and patients respond excellently to conventional treatments that include surgery, external beam radiotherapy (EBRT), radio-iodine (^131^I) and cytotoxic chemotherapies such as paclitaxel^3–5^. However, the outcomes for ATC patients are less favorable as the disease is notoriously difficult to treat^6^. The median survival of ATC patients is less than six months post diagnosis and the 5-year overall survival rate falls below 10%^7^. In cases where the primary tumor is successfully resected, patients often present with metastasized disease either at diagnosis or following initial treatment^8^. Worse still, the majority of ATC tumors are radio-iodine refractory and unresponsive to conventional chemotherapies. ATC is a highly mutated disease and several tyrosine-kinase inhibitors (TKIs) are approved for treatment of ATC, including sorafenib (nexavar) and lenvatinib (lenvima)^6^. The former is a multi-kinase inhibitor primarily targeting the vascular endothelial growth factor receptor VEGFR, platelet-derived growth factor receptor (PDGFR) and the Raf-kinases, including c-Raf and b-Raf^9,10^. Thus, ATC patients harboring BRAF-mutations (primarily the ^V600E^BRAF mutation) are eligible for treatment with sorafenib. However, high toxicity and acquired resistance to long-term treatment with sorafenib limits the efficacy of the drug.

Recently, is has been suggested that treatment with heat-shock protein 90 (HSP90) inhibitors can mitigate the acquired resistance of TKIs, including sorafenib^11–14^. HSP90 is a molecular chaperone with a plethora of downstream client proteins, many of them directly associated with malignancies. Inhibition of HSP90 results in misfolding of the client proteins, ultimately leading to cell cycle arrest and/or cell death^15^. BRAF, a frequently mutated protein in both PTC and ATC, is found among the HSP90 client proteins and the combination of sorafenib with HSP90 inhibitors has been proposed as a potent future therapeutic regimen. While several preclinical studies have assessed HSP90 inhibition in thyroid cancer, the combination with sorafenib has yet to be thoroughly explored^15,16^. A clinical study did previously investigate the combination treatment of tanespimycin (17-AAG) in combination with sorafenib in solid cancers, one of which was a PTC, with positive outcomes^17^. Nevertheless, to our knowledge the combination has not been pursued in further studies in thyroid cancer. The role of HSP90 in regard to sorafenib resistance has however been investigated in hepatocellular carcinoma (HCC) in preclinical studies, demonstrating that the combination treatment increased apoptosis both in vitro and in vivo^13^.

Onalespib (AT13387) is a potent, second-generation HSP90 inhibitor currently assessed in multiple drug-combinations, including olaparib, dabrafenib and trametinib (clinicaltrials.gov). The potency of utilizing onalespib in combination with either radiotherapy or other cytotoxic drugs has been demonstrated both in vitro and in vivo^18–22^. The specific combination of onalespib with sorafenib has yet to be investigated in thyroid cancer^16^, and subsequently the aim of this study is to determine the sensitivity of ATC and PTC cell lines to onalespib and explore the potency of the combination of onalespib and sorafenib in an in vitro and in vivo setting.

## Results

### Sorafenib and onalespib monotherapy decreases thyroid cancer cell viability, while concomitant treatment increases the efficacy significantly in BHT-101, SW1736 and MDA-T32 cells

**Figure 1:**
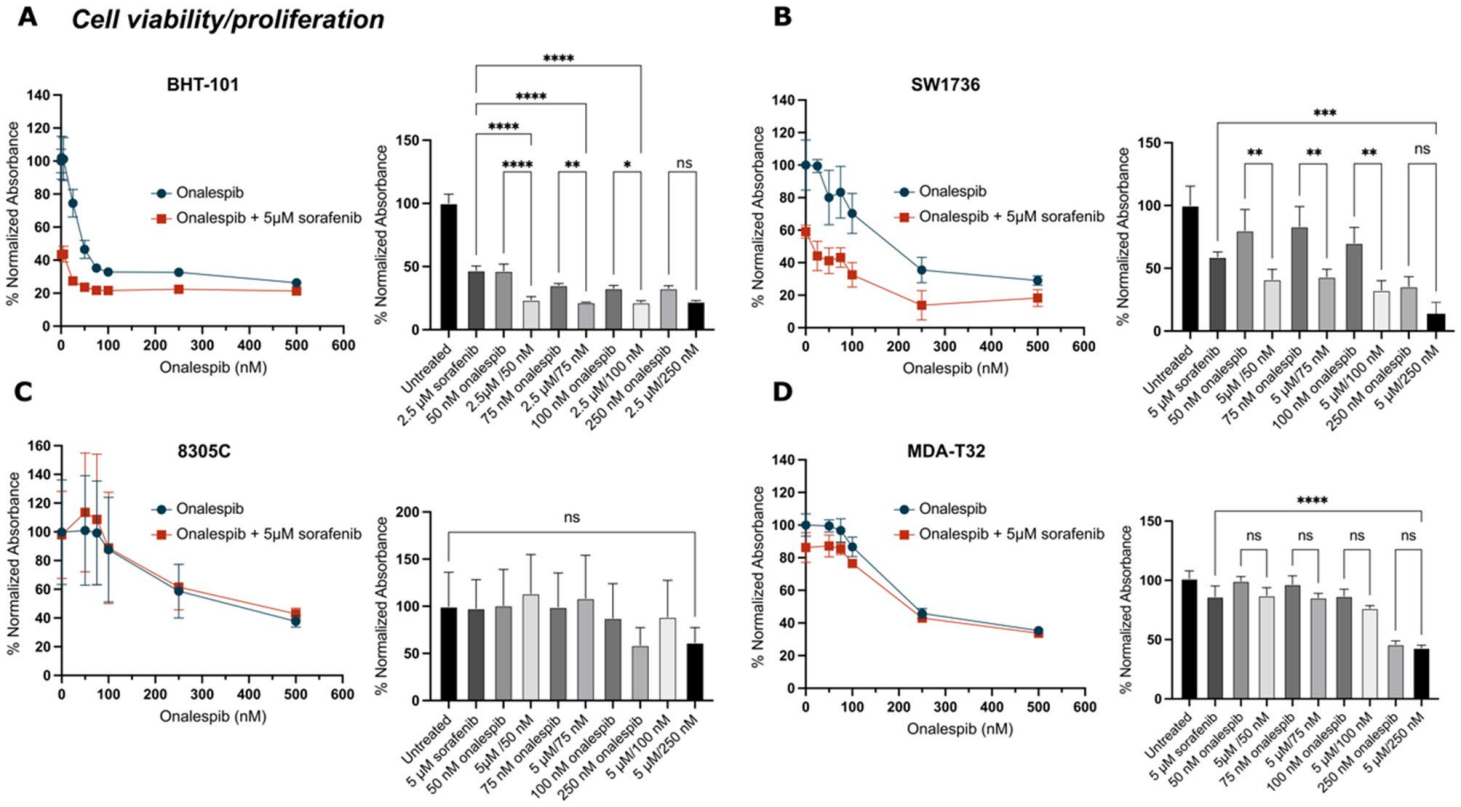
Cell viability (XTT assays) of four thyroid carcinoma cell lines: (**A**) BHT-101 (ATC), (**B**) SW1736 (ATC), C) 8305C (ATC) and D) MDA-T32 (PTC) with known BRAF V600E mutations. Cells were treated with 1 nM, 5 nM, 10 nM, 25 nM, 50 nM, 75 nM, 100 nM, 250 and 500 nM of onalespib and with a fixed dose of 2.5 μM (BHT-101) or 5 μM (SW1736, 8305C, MDA-T32) sorafenib for 72 h. Values ± SD of at least 3 independent experiments. One-way ANOVA followed by Tukey’s multiple comparisons test assessed the significance of selected combination treatments. *p < 0.05; **p < 0.01; ***p < 0.001; ****p < 0.0001.

First, the senitivity of four thyroid carcinoma cell lines to increasing doses of onalespib in combination with sorafenib was evaluated in cell viability assays. Increasing concentrations of onalespib decreased the viability in all cell lines in a concentration dependent manner (Fig. 1). The BHT-101 cells were more sensitive to lower doses of onalespib (< 100 nM) than the additional cell lines (SW1736, 8305C and MDA-T32), although sensitivity to high doses of onalespib (≥ 250 nM) was similar across all four cell lines (Fig. 1A). The BHT-101 cells were likewise highly sensitive to sorafenib, resulting in a lower dose (2.5 μM) for combination treatments with onalespib for that particular cell line (Fig. 1A). The SW1736 cells were sensitive to both onalespib and sorafenib, albeit not to the extent of the BHT-101 cells. Significant combination effects were observed in combinations using 2.5 μM sorafenib and onalespib for BHT-101 and 5 μM sorafenib and onalespib for SW1736 (Fig. 1A,B). No significant effects of sorafenib (5 μM) nor combination effects were demonstrated in the 8305C (Fig. 1C) or MDA-T32, except for the highest chosen drug concentration treatment combination (Fig. 1D).

### Sorafenib and onalespib treatment affects cell cycle distribution and induces apoptosis and necrosis

**Figure 2:**
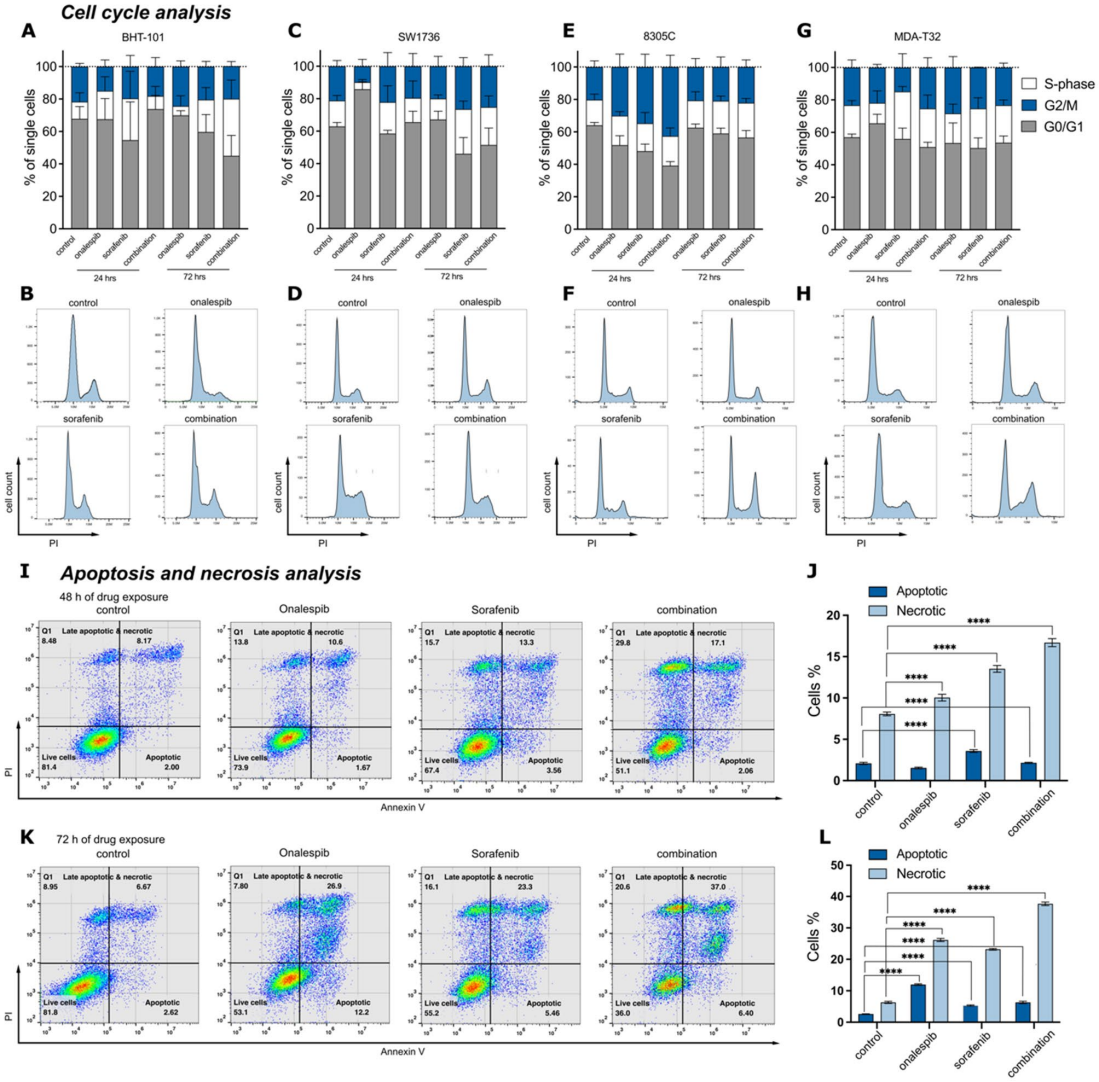
Cell cycle analysis of (**A**, **B**) BHT-101, (**C**, **D**) SW1736, (**E**, **F**) 8305C and (**G**, **H**) MD2-T32 cells measured by flow cytometry. Cells were stained with Propidium iodine (PI) 24 and 72 h after exposure to sorafenib, onalespib and the combination of both. Upper row: % of cells in G0/G1, S-phase and G2/M phase of the cell cycle. Lower row: representative histograms of the distribution. Values ± SD of 2–3 independent experiments. (**I**) Representative dot plots of apoptosis and necrosis analysis of BHT-101 cells 48 h after drug exposure. (**J**) Percentage of cells positive for PI and Annexin V staining at 48 h (**K**) Representative dot plots of apoptosis and necrosis analysis of BHT-101 cells 72 h after drug exposure. (**L**) Percentage of cells positive for PI and Annexin V staining at 72 h. Mean values ± SD. One-way ANOVA followed by Tukey’s multiple comparisons test assessed the significance of selected combination treatments. *p < 0.05; **p < 0.01; ***p < 0.001; ****p < 0.0001.

The cell cycle distribution after exposure to sorafenib, onalespib and their combination in BHT-101, SW1736, 8305C and MD2-T32 cells was studied by flow cytometry. When treated with onalespib alone, SW174 and MDA-T32 cells exited S-phase as early as 24 h after treatment, while BHT-101 showed a reduction in cells in S-phase after 72 h of treatment (Fig. 2A–H). A tendency in G1/M phase percentage increase was detected in the onalespib and combination treated groups, most dominant in 8305C cells. Sorafenib mono-treatment lead to an accumulation of cells in S-phase and reduction in G0/G1 phase in BHT-101, SW1736 and MDA-T32 cells at both investigated time points.

Furthermore, the induction of apoptosis and necrosis was investigated using flow cytometry (Fig. 2I–L and Supplementary Fig. 1). Sorafenib monotherapy significantly induced early apoptosis in BHT-101 cells 48 h and 72 h after treatment measured by Annexin V staining. Onalespib monotreatment significantly increased the number of early apoptotic cells only at the later 72 h time point (Fig. 2I–L). Combination treated cells showed an increased number of early apoptotic cells compared to the control group but not in comparison to the monotreatment groups. However, in the combination group, a drastic increase of late apoptotic and necrotic cells was observed after 48 h (16.7%), which was even more pronounced 72 h post treatment (37.65%) (Fig. 2K,L).

### Combination therapy of sorafenib and onalespib delays wound healing and reduces thyroid cancer cell migration

**Figure 3:**
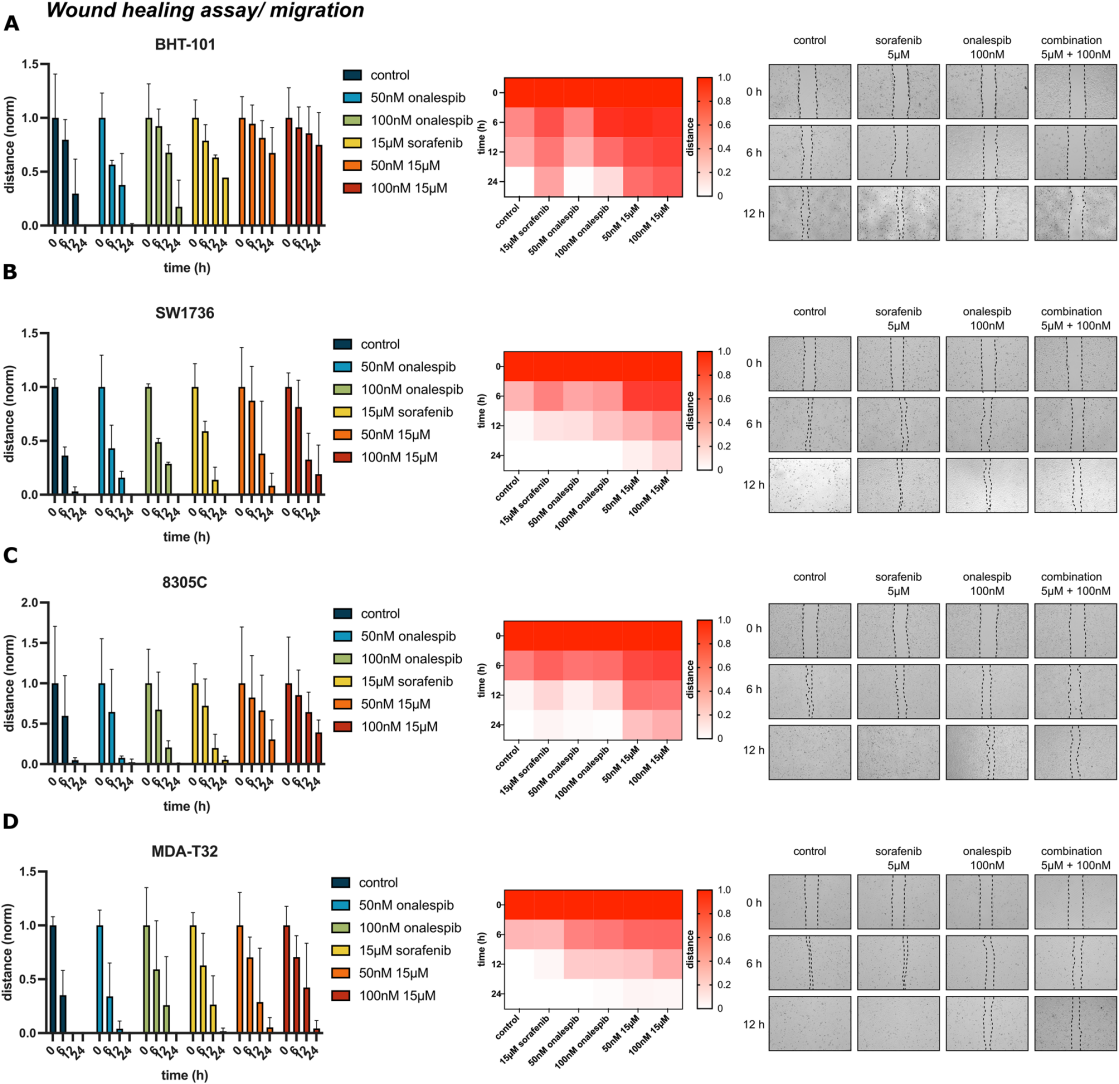
Wound healing assays of (**A**) BHT-101 (ATC), (**B**) SW1736 (ATC), (**C**) 8305C (ATC) and (**D**) MDA-T32 (PTC) cells. Cells were treated with 50 nM and 100 nM of onalespib and 15 μM sorafenib. Left column: Bar chart of migrated distance over time. Middle: heat map over migrated distance; Right: Representative images of wound/gap of control, 15 μM sorafenib, 100 nM onalespib and the combination at 0, 6, and 12 h. Values ± SD of 3 independent experiments.

Wound healing assays were performed to investigate whether treatment with onalespib and sorafenib could affect the migratory ability of BHT-101, SW1736, 8305C and MDA-T32 cells. The sensitivity of BHT-101 cells to the combination of sorafenib and onalespib previously observed in the viability assays was also demonstrated in the wound healing assays (Fig. 3). Likewise, BHT-101 cells were highly sensitive to onalespib treatments and only the untreated control group and the group with the lowest onalespib concentration (50 nM) was able to migrate into the gap at the assay endpoint (24 h, Fig. 3A). At this time point, there was a statistically significant difference between the size of the gap in the control wells and the monotreatment (100 nM onalespib and 15 μM sorafenib) as well as in all tested combination treatments (Supplementary Fig. 2). The inhibitory effect was most dominant in the combination group of 100 nM onalespib and 15 μM sorafenib, with 86% of the gap remaining open at 12 h and 75% at 24 h. In this experimental design, the SW1736 and 8305C cells showed a significantly greater response with 20% and 39% open gap at the endpoint for the combination treatment with 15 μM sorafenib and 100 nM onalespib, respectively (Fig. 3B,C, Supplementary Fig. 2). MDA-T32 cells were the most resistant to combination treatments and the remaining wound was nearly closed (4% of the original wound was still open, Fig. 3D).

### Additional treatment with onalespib synergistically potentiates sorafenib therapy

**Figure 4:**
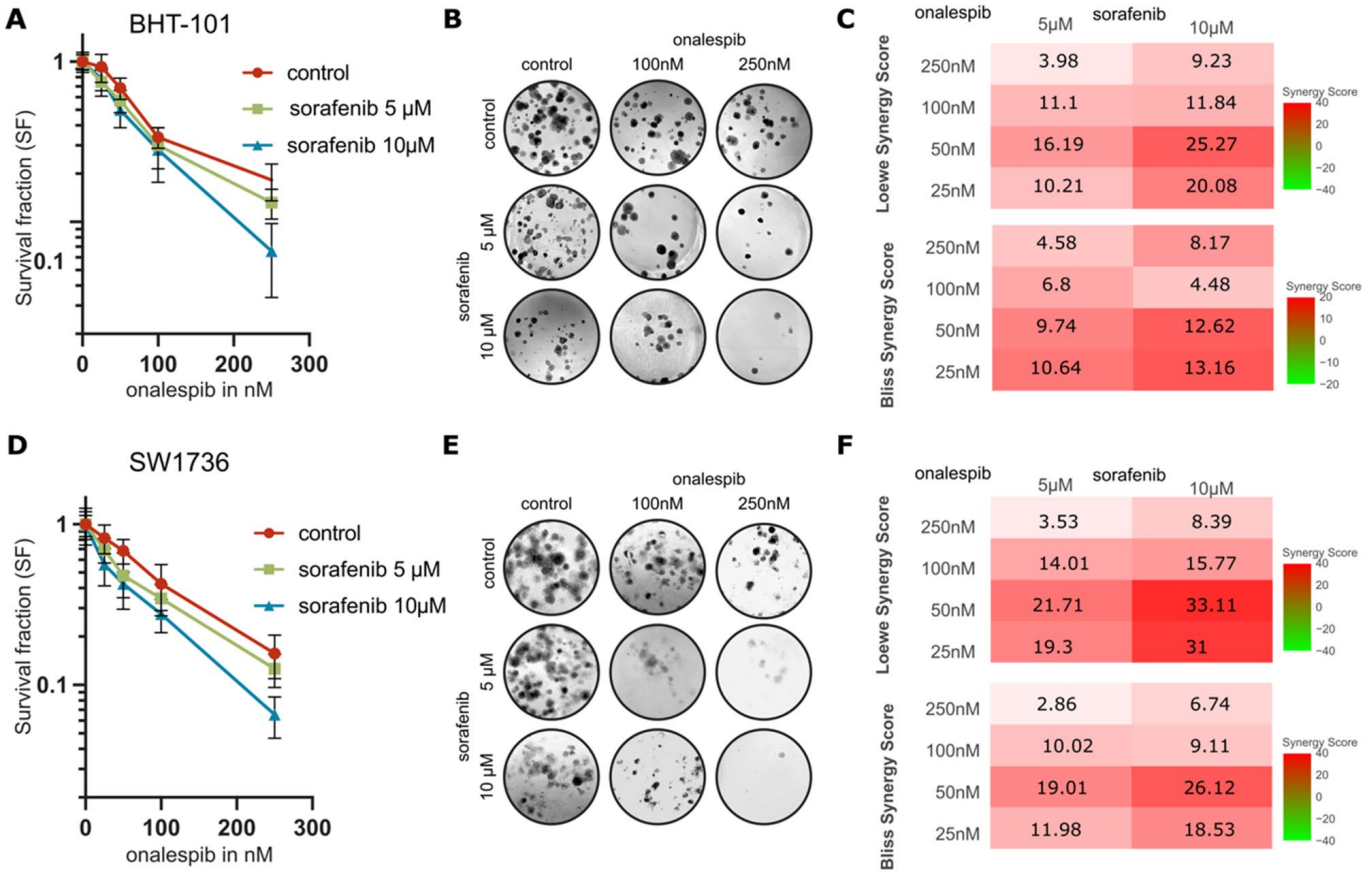
Clonogenic survival and synergy analysis (Loewe and BLISS) for concurrent sorafenib and onalespib therapy in BHT-101 and SW1736 cells. (**A**) Clonogenic survival assay of BHT-101 demonstrating sensitizing effects of the combination treatment. (**B**) Representative images of BHT-101 cells treated with sorafenib and onalespib in the indicated doses. (**C**) Loewe and BLISS score analysis of BHT-101 clonogenic survival assays. A score > 10 indicated synergy, < 10 antagonism. (**D**) Clonogenic survival assay of SW1736 demonstrating sensitizing effects of the combination treatment. (**E**) Representative images of SW1736 cells treated with sorafenib and onalespib in the indicated doses. (**F**) Loewe and BLISS score analysis of SW1736 clonogenic survival assays. A score > 10 indicated synergy, < 10 antagonism. Values ± SD of at least 3 independent experiments.

To further assess the efficacy of the combination treatment of sorafenib and onalespib, the two most responsive cell lines, BHT-101 and SW1736, were evaluated in a clonogenic survival assay to determine long-term effects (Fig. 4). Both sorafenib treatment as well as onalespib treatment reduced the cell survival in a concentration dependent manner. Statistically significant growth reduction was observed at 50, 100, and 250 nM onalespib for both BHT-101 and SW1736, and 10 μM sorafenib for BHT-101 in comparison to DMSO treated control samples, respectively. For statistical analysis see Supplementary Table 1. Long term-exposure of 100 nM onalespib alone reduced the survival fraction for BHT-101 and SW174 by 66.3% and 57.5%, respectively (Fig. 4A,D). Additional treatment with sorafenib reduced the survival further and an average of less than 6% of BHT-101 and SW174 survived combination therapy with 10 μM sorafenib and 250 nM onalespib. Synergy calculations using Loewe scoring and Bliss independence scoring demonstrated strong synergy for the combination of 10 μM sorafenib and low doses of onalespib (25 nM and 50 nM) for BHT-101 cells with Loewe and BLISS scores above 10. Synergy was also documented for combinations of 5 μM sorafenib and 25 nM onalespib. For SW1736 cells 10 μM sorafenib and onalespib concentrations ≤ 50 nM resulted in synergistic effects as well as combination of 5 μM sorafenib and ≤ 100 nM onalespib. The most potentiating effect was the combination of 10 μM sorafenib and 50 nM onalespib for BHT-101 and SW1736 with Loewe scores above 25 and 33, and BLISS scores above 12 and 26 respectively (Fig. 4C,F).

### Onalespib and orafenib treatment reduces protein expression involved in tumorigenesis, infinite replicative potential, angiogenesis, metastasis and tissue invasion

We utilized OLINK proteomic analysis to investigate the changes in protein expression profiles in BHT-101 cells treated with onalespib, sorafenib, and in combination.

**Figure 5:**
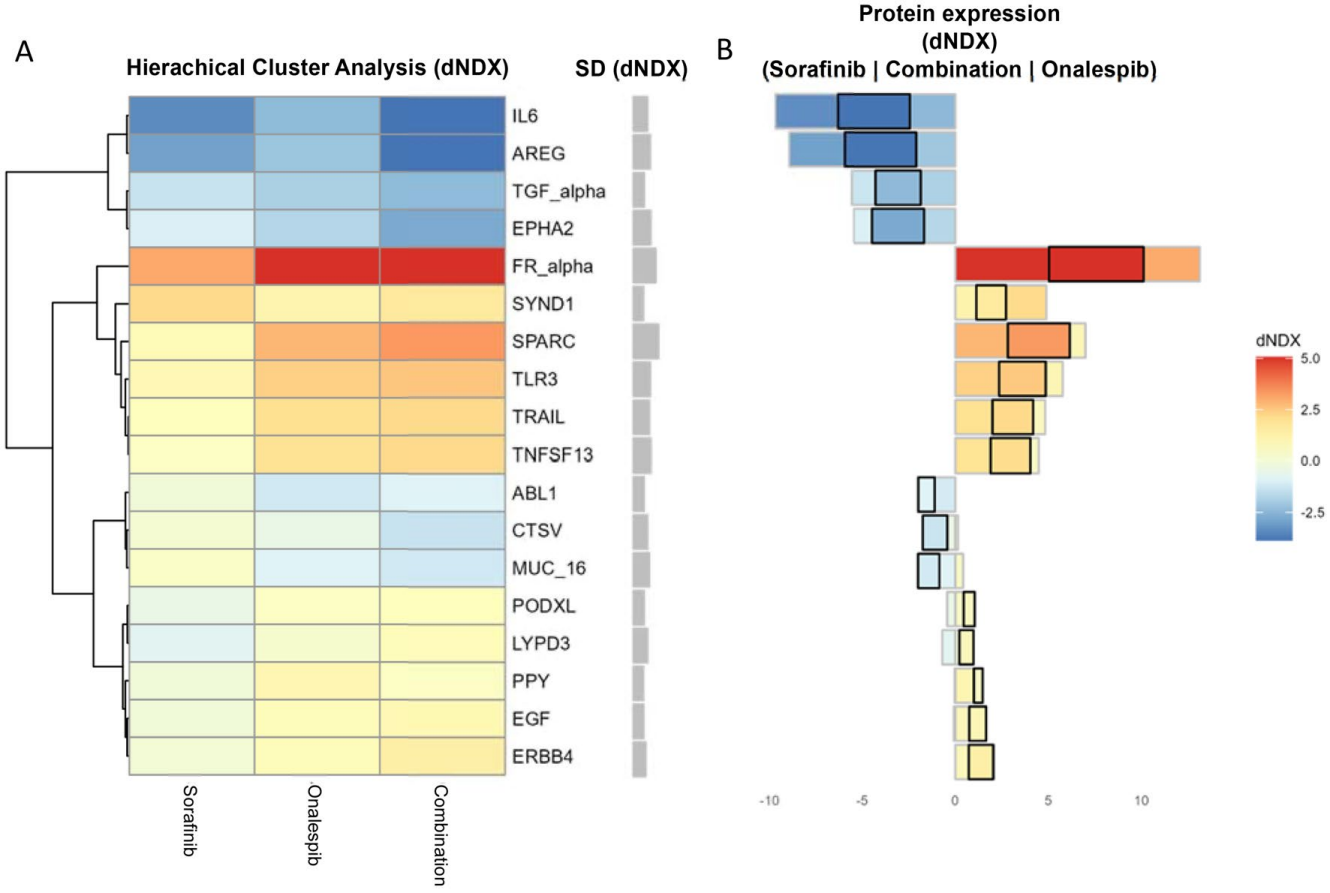
(**A**) Hierarchical cluster analysis of most significant protein expression changes of sorafenib, onalespib and combination treated BHT-101 cells, as compared to expression in treatment-free control cells (dNDX = difference in log(expression) to control). Positive values indicate higher expression than in control (red), negative values indicate lower (blue). (**B**) Absolute dNDX for each treatment compared to control, using the same scale as left. Black square indicates the combination treatment group, with sorafenib positioned to the left and onalespib on the right-hand side. The grey field in the middle shows the standard deviation in protein expression between the treatments, indicating more or less differentially expressed proteins.

The analysis revealed significant expression differences of several proteins treated with onalespib, sorafenib, and their combination as visualized in the hierarchical cluster analysis in Fig. 5 and Supplementary Fig. 3. Notably, several proteins involved in tumorigenesis, including self-sufficiency in generating growth signals, infinite replicative potential, angiogenesis, metastasis and tissue invasion, and resistance to apoptosis were significantly downregulated in monotherapy as well as in the combination treated group compared to controls (Supplementary Fig. 4). Among these proteins were several ligands of growth factor receptors, e.g. EGFR, like TNF alpha and amphiregulin (AREG) or the autocrine and paracrine growth factor IL-6. Further the tyrosine kinase receptor EphA2, which has been found to mediate invasion in thyroid cancer, was downregulated in all treatment groups. The downregulation of these oncoproteins was most pronounced in the combination treatment group compared to the monotreatment groups. The analysis further revealed upregulation of several members of the TNF superfamily like TNFRs and TRAIL which indicates the induction of cell death and apoptosis. Cathepsin V (CTSV) expression was only slightly affected by both sorafenib and onalespib treatment, however the combination therapy reduced the protein strongly. Cathepsin V is associated with poor outcome in thyroid cancer. Onalespib treatment reduced the expression of the nonreceptor tyrosine kinases ABL1, which also is associated with poor outcomes in thyroid cancer, while sorafenib treatment had no effect on the expression. On the contrary, sorafenib had slightly different effect on the tyrosine kinase ERBB4 (HER4), while the combination treatment increased the expression. In the recent years HER4 has emerged as a favorable prognostic marker for several cancers including thyroid cancer which also can act as a tumor suppressor.

### Combination therapy of sorafenib and onalespib significantly reduces tumor growth and increases median survival

**Figure 6:**
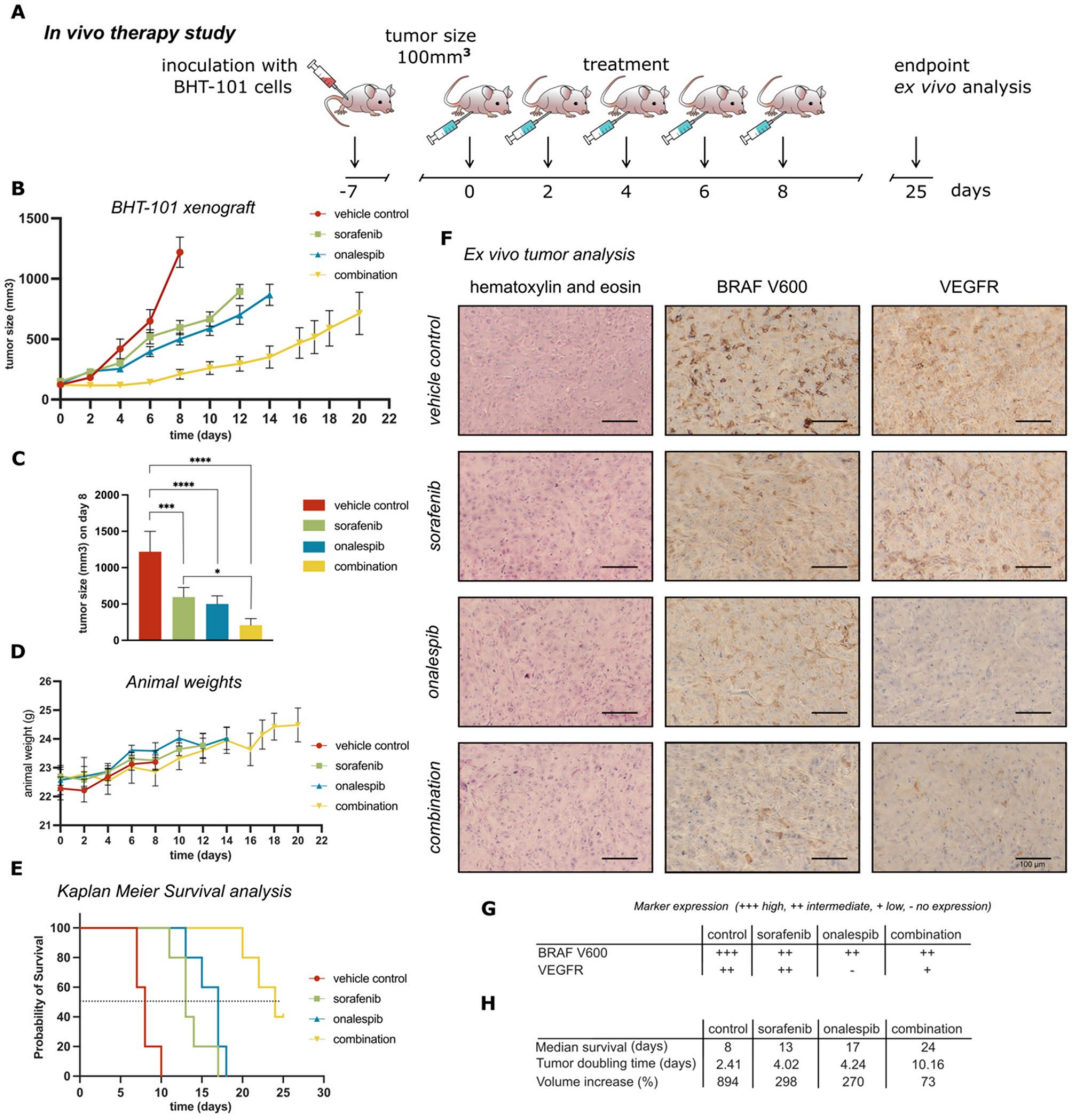
In vivo tumor growth, survival analysis and ex vivo immunohistochemistry of BHT-101 mouse xenografts. (**A**) Treatment scheme of the in vivo study. Mice received a total of five doses with sorafenib (25 mg/kg), onalespib (40 mg/kg) or the combination of them on alternative days. (**B**) Tumor growth over time for each treatment group displayed until the first animal reached the study endpoint. (**C**) Tumor growth over time for each treatment group on day 8 (last measurement before first control animal reached endpoint). One-way ANOVA followed by Tukey’s multiple comparisons test assessed the significance of selected combination treatments. *p < 0.05; **p < 0.01; ***p < 0.001; ****p < 0.0001. (**D**) Animal weights in g for each treatment group displayed until the first animal of each group reached the study endpoint. (**E**) Kaplan–Meier curves of survival of mice in the therapy study. (**F**) Representative immunohistochemistry images of BHT-101 tumors stained with hematoxylin and eosin, BRAF V600E and VEGFR-3. Size bar corresponds to 100 μm. (**G**) Change in marker expression of the different treatment groups. (**H**) Median survival of mice in the therapy study, tumor doubling time and tumor volume increase, calculated between day 0 and day 8 (where the first control animal reached the study endpoint). Data presented as mean values ± SD.

The potency of the combination of sorafenib and onalespib was evaluated in tumor-bearing mice using BHT-101 xenografts, treated as indicated in Fig. 6A. Tumor growth and animal weight was monitored every other day, shown in Fig. 6B–D. Overall, the treatments were well tolerated and no adverse effects as well as changes in body weight were observed (Fig. 6D). Sorafenib and onalespib monotherapies demonstrated similar response rates, statistically significant from control (Fig. 6C), resulting in impaired tumor growth with tumor doubling times of 4.02 days and 4.24 days respectively compared to vehicle control with a tumor doubling time of 2.41 days. The tumor growth of the combination group was likewise impaired compared to monotherapies and the doubling time increased to 10.16 days (Fig. 6C,E). This was further illustrated by a significant difference in tumor volume increase by 894% for control mice, 298% for sorafenib, 270% onalespib and 73% in the combination group. In line with these results the median survival of mice injected with sorafenib and onalespib alone increase from 13 and 17 days to 24 days for the combination group (Fig. 6E,H). Ex vivo immunohistochemistry revealed high BRAFV600 expression in control samples and intermediate expression in monotherapy and combination groups. VEGFR-3 expression was detected in control (intermediate) and sorafenib (intermediate) samples, but was downregulated in the onalespib group (Fig. 6F,G).

## Discussion

Due to the high mortality and large difference in survival between early and advanced stage of thyroid cancer, finding more effective clinical treatments for these patients remains crucial. Multi-receptor tyrosine kinase inhibitors such as sorafenib and lenvatinib are FDA-approved for the treatment of advanced thyroid cancer but none of them have shown a significant improvement in overall survival. In addition, effective strategies to prevent the emergence of TKI resistance in the clinic are currently lacking.

The exploration of sorafenib and onalespib combination treatment presented here suggests that HSP90 inhibition can be a potent therapeutic strategy in thyroid cancer. While sensitivity to both sorafenib and onalespib varied between the cell lines, sensitivity to onalespib directly correlated with more potent combination effects. The in vivo study further demonstrated the potency of the combination treatment, with significantly improved median survival between the combination group and monotherapy groups, with IHC confirming downregulation of BRAFV600 and VEGFR-3.

Narrowly targeted cancer medications such as TKIs are rapidly gaining traction in the oncology field. The advantages of targeting tumor specific mutations are plentiful and the potential seems limitless. However, TKIs are facing several uphill battles. One such battle is the acquired resistance to long-term treatment and another is the high incidence of serious side effects. For sorafenib, adverse events have been reported in up to 50% of patients, which in turn has limited the maximum tolerated doses, ultimately resulting in disappointing effects on overall survival rates. Since the approval of sorafenib, the new and more specific BRAF inhibitor dabrafenib has been assessed in clinical trials in radio-iodine refractory thyroid cancer, with lower incidence of adverse events and greater therapeutic outcomes^23^. Additionally, powerful combination effects have been demonstrated in patients receiving a combination of dabrafenib and the MEK-inhibitor trametinib, illustrating the potential of choosing a favorable combination strategy^24^.

The concept of combining HSP90 inhibition with BRAF inhibition appeared more than a decade ago and despite seemingly favorable results in early preclinical studies, this combination strategy has yet to be developed further within the thyroid cancer field. As BRAF is among the most common oncoproteins in both PTC and ATC, a strategy that interferes with BRAF, and thus the MAPK-pathway, is of great interest. While there is little doubt that sorafenib has resulted in patient benefits, the high toxicities limit the application. One strategy of circumventing the high toxicities while retaining the potent anti-tumor effects is to find a combination therapy that is able to enhance the effects without adding to the toxicity. The advantage of HSP90 inhibition is that it is not a mutational target. Rather, tumors can be more or less dependent on HSP90 and thus more or less sensitive to HSP90 inhibition^25^. In the present study, all four of the tested thyroid cancer cell lines harbor BRAFV600E mutations and should therefore respond to sorafenib. Similarly, all of the tested cell lines should be responsive to HSP90 inhibition with onalespib, although the extent of that sensitivity has yet to be explored. In short-term assays, i.e. cell viability and wound healing assays, the BHT-101 cell line was by far the most sensitive to both monotherapies and the combination of sorafenib and onalespib (Figs. 1, 2, 3). The SW1736 cells were likewise sensitive to both onalespib and sorafenib, while 8305C and MDA-T32 were resistant to sorafenib and comparatively less sensitive to onalespib, particularly at lower concentrations (< 100 nM). The sensitivity of BHT-101, moderate sensitivity of SW1736 and resistance of 8305C to sorafenib treatment are all in line with previous studies^9,26^. Similarly, the strong sensitivity of BHT-101 to HSP90 inhibition was reported by Sang et al. using ganetespib, while Kim et al. demonstrated how SW1736 was relatively resistant to treatment with the HSP90 inhibitor AUY922^27,28^. The significant decrease in viability and inhibition of wound healing capability of the combination treatment presented in this study was more distinct than in previous studies exploring the combination of sorafenib and HSP90 inhibitors in hepatocellular carcinoma cells^29^. However, the resistance to sorafenib observed in both the 8305C and MDA-T32 cell lines was not overcome by onalespib combination treatment. Interestingly, the 8305C and MDA-T32 cells were less responsive to onalespib monotherapy overall and the lack of sensitivity to the combination treatment indicates that neither the 8305C nor the MDA-T32 cell lines are dependent on HSP90.

Contrary to our expectation we only observed a small increase of cells in apoptosis in the Annexin V and Caspase 3/7 assay. However, a massive increase of necrotic cells was evident in the combination group. This observation could explain the efficacy of combination treatment observed in the XTT assay for BHT-101 cells. These results are also in line with a recent publication where the HSP90 inhibitor Ganetespib augments sorafenib efficacy via necroptosis induction in hepatocellular carcinoma^30^.

In the long-term in vitro assay (colony formation assay), the differences between the two investigated cell lines (BHT-101 and SW1736) were smaller and the responses to monotherapies and the combination therapy were similar (Fig. 4). In both cell lines, the effects of the combination of sorafenib and onalespib were synergistic at low doses of onalespib (< 100 nM). The lack of difference in sensitivity between the two cell lines was somewhat surprising, given the strong sensitivity of the BHT-101 cells in short-term assays. However, the SW1736 demonstrated sensitivity to both monotherapies and to the combination in short-term assays and it was expected that the established sensitivity translated into the colony formation assay as well.

Our OLINK proteomic analysis highlights the distinct protein expression alterations induced by onalespib, sorafenib, and their combination treatment in thyroid cancer cells. The downregulation of amphiregulin, IL-6 and EPHA2 suggests interference with pathways critical for tumor growth and progression and may explain the pronounced effect seen in the in vivo study. Also, upregulation of TLR3 has been associated with improved overall survival in thyroid cancer patients. In line with the upregulation of apoptosis-inducing TNF super-family members is the observation of upregulation of adapter molecules like FADD which can initiate apoptosis by recruitment of caspases^31^. The function of several proteins differentially expressed in the treatment groups is not fully understood as some are involved in different, sometimes ambivalent cellular processes including tumor suppression, immune modulation and cell proliferation.

FRα which was found upregulated in our study, for example, may function not only as a folate transporter, but may also confer signaling and growth advantages on malignant cells. However, a recent study has found folate receptor-positive circulating tumor cells as a biomarker to predict preferable outcome of pemetrexed-based chemotherapy in patients with advanced nsNSCLC^32^. Further, the protein SPARC (secreted protein acidic and rich in cysteine) regulates interactions between cells and their surrounding extracellular matrix, and has shown seemingly contradictory effects on tumor progression in both preclinical and clinical studies. The activity seems to be context- and cell-type-dependent. SPARC activation has shown characteristics of a tumor suppressor in many cancers including acute myeloid leukemia, neuroblastoma, carcinoma of the breast, colorectal adenocarcinoma, hepatocellular carcinoma, non-small cell and small cell lung cancer, carcinoma of the ovaries and pancreatic ductal adenocarcinoma^33^.

The full list of altered protein expression in the sorafenib, onalespib and combination treated groups (Supplementary Fig. 3) may also reveal potential therapeutic targets for future investigation. This analysis is however only a snapshot of the underlying processes and further studies are needed to elucidate the functional implications of these proteomic changes and their potential therapeutic implications for the treatment of thyroid cancer.

To further evaluate the efficacy of sorafenib and onalespib in a more clinically relevant setting, mouse xenograft studies with BHT-101 tumors were performed (Fig. 6). Both monotreatments were able to reduce the tumor growth compared to the control. The combined treatment however was most potent and produced better tumor control than either drug alone leading to a significant increase in tumor doubling time and median survival. These results mirror the excellent efficacy of the combination treatment in the in vitro studies. Since acute side effects such as weight loss were not observed at any time point in the study, demonstrating good tolerability of the combined treatment, we believe that continuous co-treatment (more than five doses) is possible and that with a prolonged treatment schedule we would have achieved a high cure rate. In the current study with a total of five doses, one of the animals in the combination group already presented with a barely detectable/measurable tumor, suggesting the possibility of full remission.

The ex vivo tumor staining indicates that onalespib treatment leads to a long-lasting downregulation of its client proteins as evident from the downregulation of VEGFR-3 at the endpoint of the in vivo study. A long duration of action of onalespib has been suggested in other cancer types, including non‐small cell lung cancer^34^. Furthermore, VEGF and its receptor are angiogenic factors that play an important role in the progression of thyroid cancer. VEGFR expression has been shown to be upregulated in ATC and PTC. Therefore, we reason that HSP90 induced inhibition of VEGFR could be one factor in the increased efficacy observed in our in vivo study. On the other hand, BRAFV600 expression was not downregulated in BHT-101 tumors, suggesting that BRAFV600 is not a client protein of HSP90, demonstrating target availability for BRAFV600 inhibitors such as sorafenib during concomitant treatment with HSP90 inhibitors.

Therefore, we conclude that the combination of BRAF V600 and HSP90 inhibition represents a novel and likely effective therapeutic option for patients with thyroid cancer, especially those that have failed previous treatments. The synergistic effects of the combination can lead to improved treatment efficacy and better clinical outcomes. However, further preclinical and clinical investigations are required to determine optimal dosing and to identify patients that would benefit most from the combination therapy.

## Materials and methods

### Thyroid cancer cell lines

8305C was purchased from Sigma Aldrich (Darmstadt, Germany) and cultured in RPMI with 20% fetal bovine serum (FBS), 1% L-glutamine and 1% antibiotics (100 IU penicillin and 100 μg/mL streptomycin) and 1% non-essential amino acids (NEAA). SW1736 was purchased from CLS Cell line service GmbH (Eppelheim, Germany) and cultured in RPMI 1640 medium with the above additives. MDA-T32 was purchased from ATCC (American Type Culture Collection, Manassas, VA, USA) and cultured in RPMI 1640 (Biowest, Nuaillé, France). The BHT-101 cell line was purchased from Deutsche Sammlung von Microorganismen und Zellkulturen (DSMZ GmbH, Braunschweig, Germany) and cultured in Dulbecco’s MEM (DMEM) with 20% FBS and 1% antibiotics (100 IU penicillin and 100 μg/mL streptomycin). All additives were acquired from Biochrom Kg, Berlin, Germany. All cells were incubated at 37 °C in an atmosphere containing 5% CO_2_.

### Cell viability assays, 2,3-bis(2-methoxy-4-nitro-5-sulfophenyl)-2H-tetrazolium-5-carboxanilide salt (XTT)

BHT-101, SW1736, 8305C and MDA-T32 cells per well were seeded in flat-bottomed 96-well plates and incubated for 48 h prior to drug incubation. For drug incubation, 0–250 nM of onalespib (Selleckchem, Germany) was incubated for 72 h as monotreatment or in combination with 2.5 μM (BHT-101) or 5 μM sorafenib (Selleckchem, Germany). Cell viability was measured and calculated according to manufacturer’s instructions of the ATCC Cell proliferation Assay Kit (ATCC, Manassas, VA, USA).

### Flow cytometry

#### Cell cycle analysis

After exposure to sorafenib (15 μM), onalespib (100 nM), or the combination thereof BHT-101, SW1736, 8305C and MDA-T32 cells were harvested and washed with ice-cold PBS followed by resuspension in 0.5 mL PBS. Cells were fixed by adding 5 mL of ice-cold 70% EtOH drop-wise and stored at − 20 °C until analysis. For analysis, the cells were centrifuged at 1200 rpm for 10 min and washed twice with ice-cold PBS. Then 0.5 mL RNase (100 μg/mL) and 100 μL of PI (50 μg/mL) was added. Cells were incubated for at least 30 min at RT in the dark before analysis using a CytoFLEX (Beckman Coulter, Krefeld, Germany). The data analysis and peaks recognition performed in FlowJoTM Software for Windows (Version 10.9 Becton, Dickinson and Company, Oregon, United States).

#### Apoptosis assay

After exposure to sorafenib (15 μM, onalespib (100 nM), or the combination thereof BHT-101 cells were harvested and washed in PBS and stained with propidium iodide (PI) and Alexa Fluor 488 annexin V (Alexa Fluor®488 Annexin V/Dead Cell Apoptosis Kit with Alexa Fluor 488 annexin V and PI for flow cytometry, ThermoFisher Scientific, Sweden) according to manufactures instructions. CellEventTM Caspase-3/7 Green Flow Cytometry Assay Kit (Thermo Fisher Scientific, Sweden) was used to analyze caspase 3/7 activity. Apoptotic cells were visualized using a CytoFLEX (Beckman Coulter, Krefeld, Germany). Obtained data were analyzed by FlowJoTM Software for Windows (Version 10.9). The number of replicates within each experimental group was four. Each experiment was repeated two times.

### Migration/wound healing

Wound healing scratch assays were performed to investigate the migratory capacity of BHT-101, SW1736, 8305C and MDA-T32 treated with sorafenib and onalespib. Cells were grown in 6 well plates to 90% confluence and incubated with sorafenib (15 μM) and onalespib (50 and 100 nM) for 24 h. Then, a scratch was made in the cell monolayer using a pink (10 μL) pipette tip, followed by media rinse and incubation with fresh cell culture media. Wound closure was monitored and photographed at 6, 12, and 24 h using a Canon EOS 700D digital camera (Canon Inc., Japan) mounted on an inverted Nikon Diaphot-TMD microscope. Migration distance was measured and analyzed using ImageJ 2.0.0 software (NIH, Bethesda, MD, USA).

### Colony formation assay

BHT-101 and SW1736 cells were seeded in 6-well plates. 24 h after seeding, cells were exposed to monotreatment of sorafenib (5 μM and 10 μM) and onalespib (25, 50, 100, 250 nM) as well as all drug combinations. 48 h after seeding, the drug containing medium was replaced and 2 mL of fresh culture medium was added. The cell cultures were incubated for 10–14 days and then ethanol fixed (4 °C), followed by staining with violet crystal solution 0.1% at room temperature. The colonies were counted manually and plating efficiency (PE) was determined by dividing the number of colonies by the number of cells initially seeded and the survival fraction (SF) was calculated by dividing the mean PE of cells exposed to the drugs by the mean PE of the untreated control. All experiments were performed in triplicates and repeated at least three times.

### Olink proteomic analysis

BHT-101 cell culture lysates were analyzed with Olinks Proximity Extension Assay using the Oncology II panel (v.7004, Olink Biosciences), measuring expression of 96 proteins. Lysates taken at 24 h post-treatment of 100 nM onalespib or 15 μM sorafenib or the combination of the two. Protein levels were expressed as normalized protein expression (NPX) on a log-scale. Values below limit of detection (LOD) were excluded from analysis (n = 29). No values were above the upper limit of quantification. All data analysis was performed with R (v4.3.1). NPX values for the treatments were adjusted by the control value to create a differential expression measure (dNDX), where positive values indicate more expression than control, and negative values indicate less. In order to analyze expression signatures between treatments hierarchical clustering was performed using the package hclust with pheatmap for visualisation, using Wards minimum variation method without scaling. The standard deviation between treatments was used to identify proteins of interest displaying high variation, indicating large differences between treatments. Proteins displaying a standard deviation of > 0.5 dNDX between treatments were selected for further analysis (n = 18), corresponding to 2^0.5 = 1.4 or 40% difference in expression. The clusterProfiler package was used to identify common functions and functional pathways for the differentially expressed proteins, using the enrichPC function and the Pathway Commons Reactome database (accessed 2023-09-01). Each treatment was repeated two times on separate occassions and the results averaged before analysis.

### Small animal therapy study

20 female Balb/c nu/nu mice (4–6 weeks of age, approx. 22.5 g) were housed under standard laboratory conditions and ad libitum access to food and water. BHT-101 cells, in serum-free media were injected subcutaneously into the right lower flank. Animal weight and size of the tumors were monitored on alternate days. Tumor diameter was measured using a digimatic caliper (Mitutoyo, Sweden) and volume was calculated as 4πabc/3 where a, b, and c were measured diameters in all dimensions. For in vivo studies, onalespib and sorafenib were dissolved in DMSO at 80 mg/ml and 50 mg/ml, respectively and further dissolved in 17.5% 2-Hydroxypropyl-beta-cyclodextrin. When tumor size reached an average of 100 mm^3^, mice received a total of 5 treatments (i.p.) with 40 mg/kg onalespib, 25 mg/kg sorafenib or combination of 40 mg/kg onalespib and 25 mg/kg sorafenib every second day. DMSO concentration was adjusted to < 10%. Control mice were injected with a mix of DMSO (10%) and 2-Hydroxypropyl-beta-cyclodextrin at the same time points as described above. All remaining animals were scarified 32 days after inoculation (25 days after first treatment). All experiments complied with current Swedish law and were performed with permission granted by the Uppsala Committee of Animal Research Ethics (permit number 10966/20). The animal study presented here was reported in accordance with ARRIVE guidelines.

### Ex vivo immunohistochemistry

Ex vivo immunohistochemistry was performed to evaluate changes in morphology as well as BRAFV600E and VEGFR expression in BHT-101 tumors. Mice bearing BHT-101 tumors were treated with onalespib and/or sorafenib as previously described (n = 12). When tumors reached a size of 1000 mm^3^ or day 25 (endpoint of experiment), tumors were collected and fixed in 4% buffered formalin and transferred to 70% ethanol. Tumor tissues were paraffin embedded, sectioned, and deparaffinized. Immunohistochemical stainings were performed on FFPE sections using fully automated protocols (DAKO Autostainer Link48) and Envision FLEX, high pH detection kit from DAKO #K8000. The following antibodies were used: BRAF V600E (Sigma Aldrich, #SAB560047, rabbit monoclonal, clone RM8, diluted 1:2000) and VEGFR3 (abcam, #ab27278, polyclonal, diluted 1:400). Immunohistochemical sections were manually scored according to staining intensity (negative −, weak +, moderate ++, or strong  +++).

### Statistical analysis

Data received from the experiments wad processed in Microsoft Office Excel for Mac Version 16.71 and all graphs have been plotted in GraphPad Prism 9 for Mac OS X. Results of the viability, proliferation, migration assays and in vivo analysis were evaluated by one-way ANOVA with Tukey's posttest in GraphPad Prism 9, where a P-value ≤ 0.05 was considered to indicate a statistically significant difference. Data are presented as the means ± standard deviation (SD).

### Synergy calculations

#### In vitro synergy calculations

The SynergyFinder webpage (https://synergyfinder.org, visited March 2023) was used for synergy calculations on the clonogenic survival and migration assay data, and yielded dose–response curves, IC50 for each individual treatment, as well as ZIP/Loewe/Bliss synergy scores^35^.

### In vivo synergy calculations

The combination effects of sorafenib and onalespib on tumor growth were assessed according to the BLISS independence method on day 8, when no animal had yet reached the endpoint (1000 mm^3^). The combination Index (CI) was calculated as described earlier^35^. In short: E_AB_ = E_A_ + E_B_(1 − E_A_), where E_A_ and E_B_ represent the observed effects of drug A and B, and E_AB_ the effect of drug A combined with drug B. CI = (E_A_ + E_B_ − E_A_E_B_)/E_AB_ being indicative of synergy (CI < 1), antagonism (CI > 1) or additivity (CI = 1).

### Tumor doubling time calculations

The tumor doubling time was calculated using the modified Schwartz formula: tumor doubling time = [ln2 × ∆T]/[ln (X2/X1)], X1 = the tumor size at day 0, initial treatment day (average tumor size 100 mm^3^), X2 = tumor size at day 8 (last day before some animals reached endpoint (1000 mm^3^). ∆T = time (in days) between the two measurements.

## Supplementary Information

Supplementary Figures.

Supplementary Table S1.

## Data Availability

Available on request, please contact Associate Professor Diana Spiegelberg, diana.spiegelberg@surgsci.uu.se.
