# Author Correction: Combination therapy of tyrosine kinase inhibitor sorafenib with the HSP90 inhibitor onalespib as a novel treatment regimen for thyroid cancer

**DOI:** 10.1038/s41598-025-13541-y

**Published:** 2025-07-29

**Authors:** Anja Charlotte Lundgren Mortensen, Hanna Berglund, Mehran Hariri, Eleftherios Papalanis, Christer Malmberg, Diana Spiegelberg

**Affiliations:** 1 Department of Immunology, Genetics and Pathology, Uppsala University, Uppsala, Sweden; 2 Department of Molecular Medicine and Surgery, Karolinska Institutet, Stockholm, Sweden; 3 Department of Medical Sciences, Uppsala University, Uppsala, Sweden; 4 Department of Surgical Sciences, Uppsala University, Uppsala, Sweden

Correction to: *Scientific Reports* 10.1038/s41598-023-43486-z, published online 06 October 2023

The original version of this Article contained an error in Figure 3, where due to a mix-up in labelling the results, the 0 h untreated images for MDA-T32 were duplicated and rearranged from the BHT-101 images shown in Figure 3A, which led to incorrect results shown in Figure 3D. The original Figure 3 and accompanying legend appear below.

**Fig. 3:**
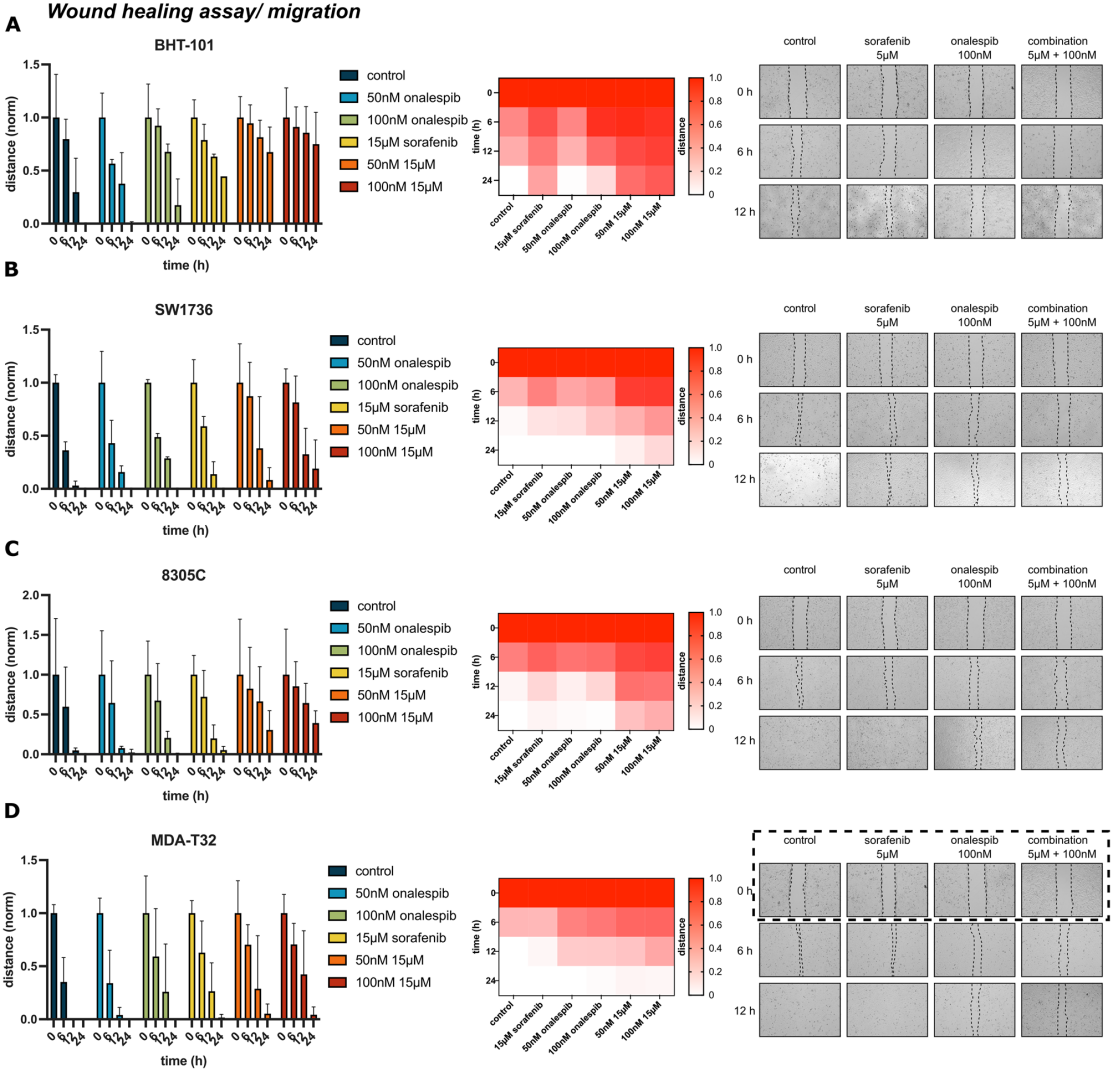
Wound healing assays of (**A**) BHT-101 (ATC), (**B**) SW1736 (ATC), (**C**) 8305C (ATC) and (**D**) MDA-T32 (PTC) cells. Cells were treated with 50 nM and 100 nM of onalespib and 15 μM sorafenib. Left column: Bar chart of migrated distance over time. Middle: heat map over migrated distance; Right: Representative images of wound/gap of control, 15 μM sorafenib, 100 nM onalespib and the combination at 0, 6, and 12 h. Values ± SD of 3 independent experiments.

The original Article has been corrected.

