# Supplementary material for: Combination therapy of tyrosine kinase inhibitor sorafenib with the HSP90 inhibitor onalespib as a novel treatment regimen for thyroid cancer
Source: Sci Rep. 2023 Oct 6;13:16844. doi: 10.1038/s41598-023-43486-z (PMC10558458; doi:10.1038/s41598-023-43486-z)
Supplement: Supplementary file 1 — Supplementary Figures. [file 41598_2023_43486_MOESM1_ESM.docx]

**Supplementary Figures**

**
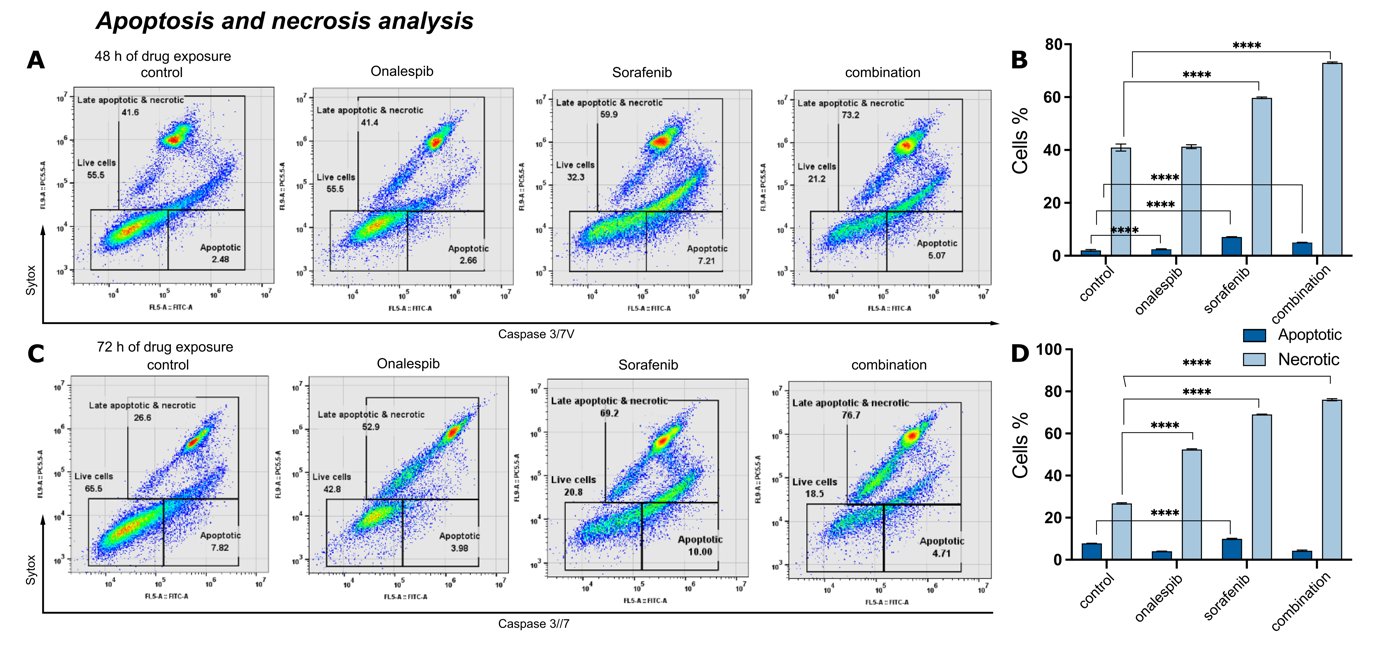
**

Supplementary Figure 1. Caspase 3/7 analysis. A) Representative dot plots of apoptosis and necrosis analysis of BHT-101 cells 48h after drug exposure. J) Percentage of cells positive for Sytox och Caspase3/7 at 48 h C) Representative dot plots of apoptosis and necrosis analysis of BHT-101 cells 72h after drug exposure. D) Percentage of cells positive for Sytox och Caspase3/7 at 72h. One-way ANOVA followed by Tukey’s multiple comparisons test assessed the significance of selected combination treatments. *p < 0.05, **p < 0.01, ***p < 0.001, ****p < 0.0001

**
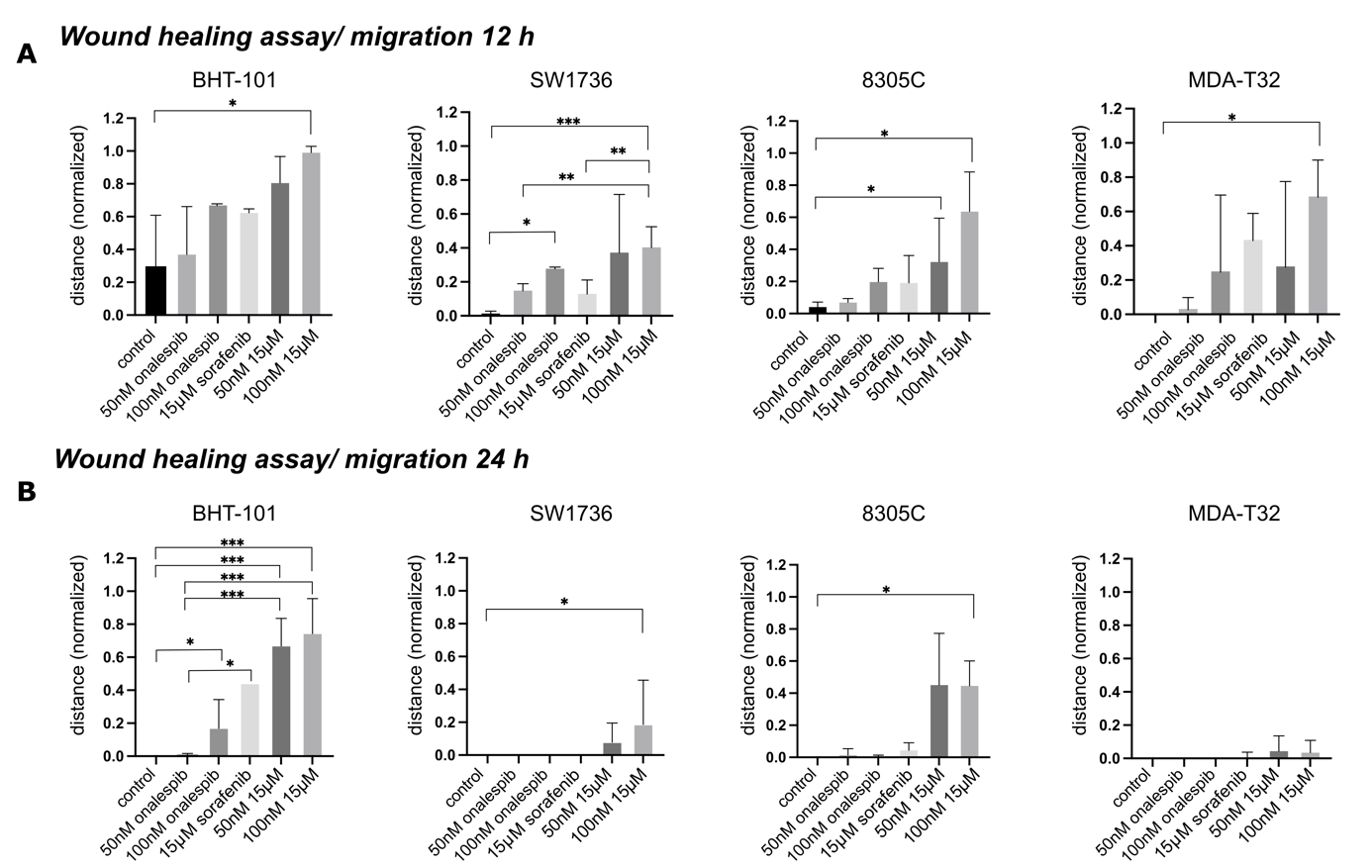
**

Supplementary Figure 2. Wound healing/migration assay for BHT-101 (ATC), SW1736 (ATC), 8305C (ATC) and MDA-T32 (PTC) cells A) 12h after induction of the wound and B) 24 h after induction of the wound. Data presented as mean values ± SD for 3 independent experiments. One-way ANOVA followed by Tukey’s multiple comparisons test assessed the significance of treatments. *p < 0.05, **p < 0.01, ***p < 0.001, ****p < 0.0001.

**
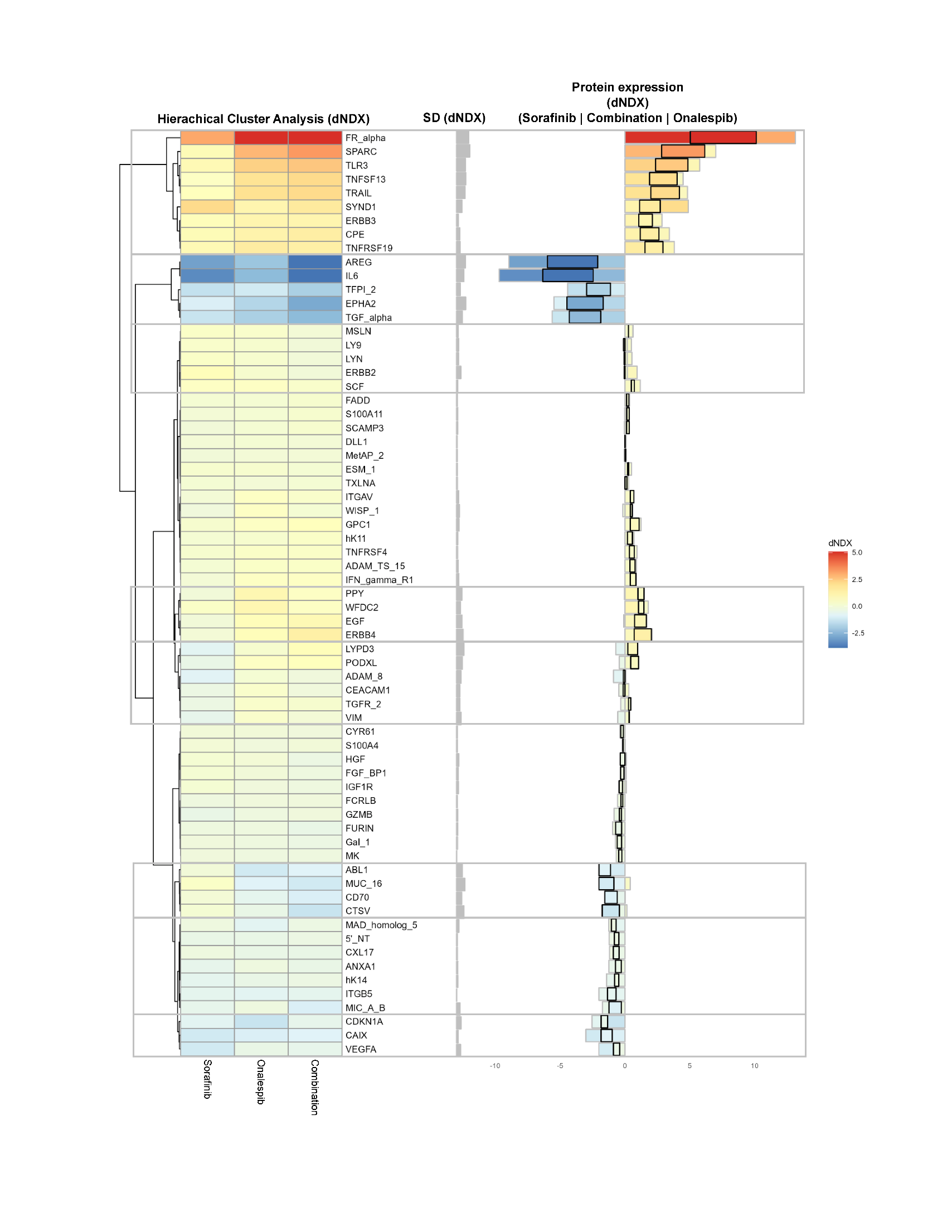
**

Supplementary Figure 3. Left: Hierarchical cluster analysis of protein expression of sorafenib, onalespib and combination treated BHT-101 cells, as compared to expression in treatment-free control cells (dNDX = difference in log_2_(expression) to control). Positive values indicate higher expression than in control (red), negative values indicate lower (blue). Right: Absolute dNDX for each treatment compared to control, using the same scale as left. Black square indicates the combination treatment group, with Sorafenib positioned to the left and Onalespib on the right-hand side. Middle bar in grey: The standard deviation between treatments, where a large standard deviation indicates differentially expressed proteins of interest.The grey boxes delineate divergent clusters of interest of proteins with similar expression patterns. Mean values of 2 independent experiments.


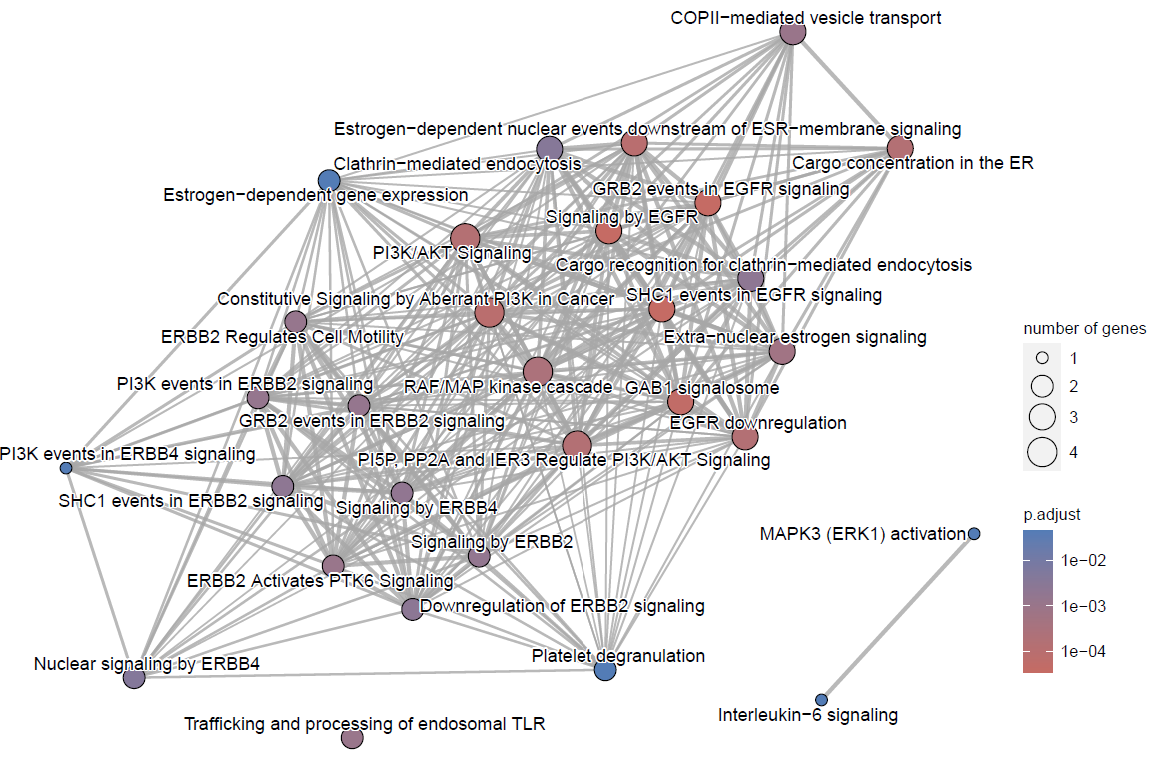


*Supplementary figure 4. Functional similarity of the differentially expressed proteins between the treatment groups. Similarities were assessed by using the Pathway Commons Reactome database, and curated based on function.*
