# Supplementary material for: Combination therapy of tyrosine kinase inhibitor sorafenib with the HSP90 inhibitor onalespib as a novel treatment regimen for thyroid cancer
Source: Sci Rep. 2023 Oct 6;13:16844. doi: 10.1038/s41598-023-43486-z (PMC10558458; doi:10.1038/s41598-023-43486-z)
Supplement: Supplementary file 2 — Supplementary Table S1. [file 41598_2023_43486_MOESM2_ESM.docx]

Supplementary table 1. Clonogenic survival for BHT-101 and SW1736 cells. One-way ANOVA followed by Tukey’s multiple comparisons test assessed the significance of treatments. *p < 0.05, **p < 0.01, ***p < 0.001, ****p < 0.0001.

| Tukey's multiple comparisons test | BHT-101 | SW1736 |
| --- | --- | --- |
| control vs. Sorafenib 5 µM | ns | ns |
| control vs. Sorafenib 10µM | **** | ns |
| control vs. Onalespib 25 nM | ns | ns |
| control vs. Onalespib 50 nM | **** | **** |
| control vs. Onalespib 100 nM | **** | **** |
| control vs. Onalespib 250 nM | **** | **** |
| control vs. Sorafenib 5 µM + Onalespib 25 nM | **** | **** |
| control vs. Sorafenib 5 µM + Onalespib 50 nM | **** | **** |
| control vs. Sorafenib 5 µM + Onalespib 100 nM | **** | **** |
| control vs. Sorafenib 5 µM + Onalespib 250 nM | **** | **** |
| control vs. Sorafenib 10 µM + Onalespib 25 nM | **** | **** |
| control vs. Sorafenib 10 µM + Onalespib 50 nM | **** | **** |
| control vs. Sorafenib 10 µM + Onalespib 100 nM | **** | **** |
| control vs. Sorafenib 10 µM + Onalespib 250 nM | **** | **** |
| Sorafenib 5 µM vs. Sorafenib 10µM | ns | ns |
| Sorafenib 5 µM vs. Onalespib 25 nM | ns | ns |
| Sorafenib 5 µM vs. Onalespib 50 nM | **** | ** |
| Sorafenib 5 µM vs. Onalespib 100 nM | **** | **** |
| Sorafenib 5 µM vs. Onalespib 100 nM | **** | **** |
| Sorafenib 5 µM vs. Sorafenib 5 µM + Onalespib 25 nM | **** | *** |
| Sorafenib 5 µM vs. Sorafenib 5 µM+ Onalespib 50 nM | **** | **** |
| Sorafenib 5 µM vs. Sorafenib 5 µM + Onalespib 100 nM | **** | **** |
| Sorafenib 5 µM vs. Sorafenib 5 µM + Onalespib 250 nM | **** | **** |
| Sorafenib 5 µM vs. Sorafenib 0 µM + Onalespib 25 nM | **** | **** |
| Sorafenib 5 µM vs. Sorafenib 10 µM + Onalespib 50 nM | **** | **** |
| Sorafenib 5 µM vs. Sorafenib 10 µM + Onalespib 100 nM | **** | **** |
| Sorafenib 5 µM vs. Sorafenib 10 µM + Onalespib 250 nM | **** | **** |
| Sorafenib 10µM vs. Onalespib 25 nM | ns | ns |
| Sorafenib 10µM vs. Onalespib 50 nM | ns | ns |
| Sorafenib 10µM vs. Onalespib 100 nM | **** | **** |
| Sorafenib 10µM vs. Onalespib 250 nM | **** | **** |
| Sorafenib 10µM vs. Sorafenib 5 µM + Onalespib 25 nM | ns | * |
| Sorafenib 10µM vs. Sorafenib 5 µM + Onalespib 50 nM | **** | **** |
| Sorafenib 10µM vs. Sorafenib 5 µM + Onalespib 100 nM | **** | **** |
| Sorafenib 10µM vs. Sorafenib 5 µM + Onalespib 250 nM | **** | **** |
| Sorafenib 10µM vs. Sorafenib 10 µM + Onalespib 25 nM | **** | **** |
| Sorafenib 10µM vs. Sorafenib 10 µM + Onalespib 50 nM | **** | **** |
| Sorafenib 10µM vs. Sorafenib 10 µM + Onalespib 100 nM | **** | **** |
| Sorafenib 10µM vs. Sorafenib 10 µM + Onalespib 250 nM | **** | **** |
| Onalespib 25 nM vs. Onalespib 50 nM | **** | ns |
| Onalespib 25 nM vs. Onalespib 100 nM | **** | **** |
| Onalespib 25 nM vs. Onalespib 250 nM | **** | **** |
| Onalespib 25 nM vs. Sorafenib 5 µM + Onalespib 25 nM | **** | ns |
| Onalespib 25 nM vs. Sorafenib 5 µM + Onalespib 50 nM | **** | **** |
| Onalespib 25 nM vs. Sorafenib 5 µM + Onalespib 100 nM | **** | **** |
| Onalespib 25 nM vs. Sorafenib 5 µM + Onalespib 250 nM | **** | **** |
| Onalespib 25 nM vs. Sorafenib 10 µM + Onalespib 25 nM | **** | *** |
| Onalespib 25 nM vs. Sorafenib 10 µM + Onalespib 50 nM | **** | **** |
| Onalespib 25 nM vs. s10 100 | **** | **** |
| Onalespib 25 nM vs. Sorafenib 10 µM + Onalespib 250 nM | **** | **** |
| Onalespib 50 nM vs. Onalespib 100 nM | **** | ** |
| Onalespib 50 nM vs. Onalespib 250 nM | **** | **** |
| Onalespib 50 nM vs. Sorafenib 5 µM + Onalespib 25 nM | ns | ns |
| Onalespib 50 nM vs. Sorafenib 5 µM + Onalespib 50 nM | *** | ** |
| Onalespib 50 nM vs. Sorafenib 5 µM + Onalespib 100 nM | **** | **** |
| Onalespib 50 nM vs. Sorafenib 5 µM + Onalespib 250 nM | **** | **** |
| Onalespib 50 nM vs. Sorafenib 10 µM + Onalespib 25 nM | ns | ns |
| Onalespib 50 nM vs. Sorafenib 10 µM + Onalespib 50 nM | **** | **** |
| Onalespib 50 nM vs. Sorafenib 10 µM + Onalespib 100 nM | **** | **** |
| Onalespib 50 nM vs. Sorafenib 10 µM + Onalespib 250 nM | **** | **** |
| Onalespib 100 nM vs. Onalespib 250 nM | ns | ** |
| Onalespib 100 nM vs. Sorafenib 5 µM + Onalespib 25 nM | **** | * |
| Onalespib 100 nM vs. Sorafenib 5 µM + Onalespib 50 nM | **** | ns |
| Onalespib 100 nM vs. Sorafenib 5 µM + Onalespib 100 nM | ns | ns |
| Onalespib 100 nM vs. Sorafenib 5 µM + Onalespib 250 nM | ** | **** |
| Onalespib 100 nM vs. Sorafenib 10 µM + Onalespib 25 nM | **** | ns |
| Onalespib 100 nM vs. Sorafenib 10 µM + Onalespib 50 nM | * | ns |
| Onalespib 100 nM vs. orafenib 10 µM + Onalespib 100 nM | ns | ns |
| Onalespib 100 nM vs. Sorafenib 10 µM + Onalespib 250 nM | **** | **** |
| Onalespib 250 nM vs. Sorafenib 5 µM + Onalespib 25 nM | **** | **** |
| Onalespib 250 nM vs. Sorafenib 5 µM + Onalespib 50 nM | **** | *** |
| Onalespib 250 nM vs. Sorafenib 5 µM + Onalespib 100 nM | ns | ns |
| Onalespib 250 nM vs. Sorafenib 5 µM + Onalespib 250 nM | ns | ns |
| Onalespib 250 nM vs. Sorafenib 10 µM + Onalespib 25 nM | **** | **** |
| Onalespib 250 nM vs. Sorafenib 10 µM + Onalespib 50 nM | **** | ns |
| Onalespib 250 nM vs. orafenib 10 µM + Onalespib 100 nM | ns | ns |
| Onalespib 250 nM vs. Sorafenib 10 µM + Onalespib 250 nM | ns | ns |
| Sorafenib 5 µM + Onalespib 25 nM vs. Sorafenib 5 µM + Onalespib 50 nM | **** | ns |
| Sorafenib 5 µM + Onalespib 25 nM vs. Sorafenib 5 µM + Onalespib 100 nM | **** | **** |
| Sorafenib 5 µM + Onalespib 25 nM vs. Sorafenib 5 µM + Onalespib 250 nM | **** | **** |
| Sorafenib 5 µM + Onalespib 25 nM vs. Sorafenib 10 µM + Onalespib 25 nM | ns | ns |
| Sorafenib 5 µM + Onalespib 25 nM vs. Sorafenib 10 µM + Onalespib 50 nM | **** | **** |
| Sorafenib 5 µM + Onalespib 25 nM vs. Sorafenib 10 µM + Onalespib 100 nM | **** | **** |
| Sorafenib 5 µM + Onalespib 25 nM vs. Sorafenib 10 µM + Onalespib 250 nM | **** | **** |
| Sorafenib 5 µM + Onalespib 50 nM vs. Sorafenib 5 µM + Onalespib 100 nM | **** | ns |
| Sorafenib 5 µM + Onalespib 50 nM vs. Sorafenib 5 µM + Onalespib 250 nM | **** | **** |
| Sorafenib 5 µM + Onalespib 50 nM vs. Sorafenib 10 µM + Onalespib 25 nM | ns | ns |
| Sorafenib 5 µM + Onalespib 50 nM vs. Sorafenib 10 µM + Onalespib 50 nM | ns | ns |
| Sorafenib 5 µM + Onalespib 50 nM vs. Sorafenib 10 µM + Onalespib 100 nM | **** | ns |
| Sorafenib 5 µM + Onalespib 50 nM vs. Sorafenib 10 µM + Onalespib 250 nM | **** | **** |
| Sorafenib 5 µM + Onalespib 100 nM vs. Sorafenib 5 µM + Onalespib 250 nM | ns | ns |
| Sorafenib 5 µM + Onalespib 100 nM vs. Sorafenib 10 µM + Onalespib 25 nM | **** | * |
| Sorafenib 5 µM + Onalespib 100 nM vs. Sorafenib 10 µM + Onalespib 50 nM | *** | ns |
| Sorafenib 5 µM + Onalespib 100 nM vs. Sorafenib 10 µM + Onalespib 100 nM | ns | ns |
| Sorafenib 5 µM + Onalespib 100 nM vs. Sorafenib 10 µM + Onalespib 250 nM | ** | * |
| Sorafenib 5 µM + Onalespib 250 nM vs. Sorafenib 10 µM + Onalespib 25 nM | **** | **** |
| Sorafenib 5 µM + Onalespib 250 nM vs. Sorafenib 10 µM + Onalespib 50 nM | **** | * |
| Sorafenib 5 µM + Onalespib 250 nM vs. Sorafenib 10 µM + Onalespib 100 nM | ns | ns |
| Sorafenib 5 µM + Onalespib 250 nM vs. Sorafenib 10 µM + Onalespib 250 nM | ns | ns |
| Sorafenib 10 µM + Onalespib 25 nM vs. Sorafenib 10 µM + Onalespib 50 nM | *** | ns |
| Sorafenib 10 µM + Onalespib 25 nM vs. Sorafenib 10 µM + Onalespib 100 nM | **** | ** |
| Sorafenib 10 µM + Onalespib 25 nM vs. Sorafenib 10 µM + Onalespib 250 nM | **** | **** |
| Sorafenib 10 µM + Onalespib 50 nM vs. Sorafenib 10 µM + Onalespib 100 nM | **** | ns |
| Sorafenib 10 µM + Onalespib 50 nM vs. Sorafenib 10 µM + Onalespib 250 nM | **** | ** |
| Sorafenib 10 µM + Onalespib 100 nM vs. Sorafenib 10 µM + Onalespib 250 nM | ** | * |
